# Aboriginal Community Controlled Health Services: An Act of Resistance against Australia’s Neoliberal Ideologies

**DOI:** 10.3390/ijerph191610058

**Published:** 2022-08-15

**Authors:** Brianna F. Poirier, Joanne Hedges, Gustavo Soares, Lisa M. Jamieson

**Affiliations:** Australian Research Centre for Population Oral Health, Adelaide Dental School, University of Adelaide, Adelaide 5000, Australia

**Keywords:** Indigenous health, Aboriginal Community Controlled Health Services, neoliberalism, resistance, self-determination

## Abstract

The individualistic and colonial foundations of neoliberal socio-political ideologies are embedded throughout Australian health systems, services, and discourses. Not only does neoliberalism undermine Aboriginal and Torres Strait Islander collectivist values by emphasizing personal autonomy, but it has significant implications for Aboriginal and Torres Strait Islander health. Aboriginal Community Controlled Health Services (ACCHS) operate within Community-oriented holistic understandings of well-being that contradict neoliberal values that Western health services operate within. Therefore, this paper aims to explore the role of ACCHS in resisting the pervasive nature of neoliberalism through the prioritization of self-determination for Aboriginal and Torres Strait Islander Peoples. Utilizing a critical evaluative commentary, we reflect on Aboriginal political leadership and advocacy during the 1970s and 1980s and the development of neoliberalism in Australia in the context of ACCHS. Community controlled primary health services across Australia are the only remaining government-funded and Aboriginal-controlled organizations. Not only do ACCHS models resist neoliberal ideologies of reduced public expenditure and dominant individualistic models of care, but they also incontrovertibly strengthen individual and Community health. ACCHS remain the gold standard model by ensuring Aboriginal and Torres Strait Islander rights to the self-determination of health in accordance with the United Nations Declaration of the Rights of Indigenous Peoples.

## 1. Introduction

The existence of Aboriginal Community Controlled Health Services (ACCHS) in Australia is inherently political. The origins of the Community controlled sector are intrinsically linked with Australia’s Black Power movement [1]. The Black Power movement gained traction after the 1965 Australian Freedom Rides and has been defined as a “loose coalition of young Indigenous activists” [2]. Black Power leaders in Australia expanded on previous iterations of Aboriginal and Torres Strait Islander activism while focusing on three principles: self-determination, land rights, and economic independence. The Black Power movement harnessed a grassroots resistance, advanced critiques of Australian racism, and promoted cultural pride [1]. During this time, ACCHS were established by supporters and leaders of the Black Power movement to overcome the structural racism embedded within Australia’s mainstream health system and to provide an immediate solution to the poor health experiences of Aboriginal and Torres Strait Islander Peoples [3]. The mainstream health services available at this time promoted exclusionary and discriminatory practices, including the denial of service provision to Aboriginal and Torres Strait Islander Peoples [4]. The Black Power movement provided the leadership to identify and the platform from which to combat the structural racism present in Australia’s health system; the need for an alternative health system for Aboriginal and Torres Strait Islander communities was made clear by Black Power leaders. In alignment with the collectivist values of Aboriginal and Torres Strait Islander Peoples, leaders at this time turned to the communities to address this need. The first ACCHS opened in 1971, predating the Alma Ata statement and declaration of Community primary healthcare principles by seven years, which further demonstrates the longevity of Aboriginal and Torres Strait Islander leadership in Community-owned healthcare models [5].

Importantly, ACCHS were never simply healthcare providers but fundamentally political organizations founded by Aboriginal and Torres Strait Islander Peoples “in an attempt to regain control over their lives after almost two centuries of oppression and disempowerment” [3]. The establishment of ACCHS was done without government assistance or funding. Communities and supporters provided premises for services, renovated sites, and provided transportation for those wanting to access services, while doctors worked without pay in many cases and used their own equipment [3]. To outsiders, these organizations may have appeared insignificant, but they were a critical articulation of Aboriginal and Torres Strait Islander autonomy and self-determination, where health services were conceived, designed, established, and controlled by Aboriginal and Torres Strait Islander communities.

The rise of neoliberalism in Australia, and indeed globally, paralleled the development of ACCHS. Neoliberalism is the dominant political, economic, and social orientation and philosophy of Australia and most OECD (Organization for Economic Co-operation and Development) countries today. The uptake of neoliberal ideologies of both the major political parties in Australia resulted in the introduction of programs that prioritized economic liberalization, reduced trade protections, deregulated markets, and privatized government corporations [6]. In Australia, and indeed globally, neoliberalism continues to disproportionately benefit the middle and upper classes while considerably increasing economic and health inequities [7,8,9]. Neoliberalism has pervasive impacts on Aboriginal and Torres Strait Islander well-being through observable modifications to policies that structure social resources [10], as well as insidious processes of internalization [11] and more covert influences articulated as generative mechanisms [11]. Scholarship is increasingly acknowledging the relationship between political economies and health outcomes and rightfully attributing experiences of poor health, including Aboriginal and Torres Strait Islander experiences, to the colonial and neoliberal models of government that continue to marginalize Indigenous Peoples [11,12,13].

Previous works have identified the need to amplify Indigenous resistance to neoliberalism and associated assertions of self-determination, indicating that failing to do so is to seriously restrict transformative action and the potential for a world order that commands health equity [12,14]. In consideration of the colonial and paternalistic approach to Aboriginal and Torres Strait Islander affairs in Australia and the neoliberal ideologies of privatization, reduced expenditure on public infrastructure, and personal autonomy; we, therefore, argue that Aboriginal Community Controlled Health Services successfully operate in resistance to Australian neoliberal ideologies while maintaining the right to self-determination for Aboriginal and Torres Strait Islander Peoples.

## 2. The Neoliberal Disempowerment of Aboriginal and Torres Strait Islander Self-Determination

The human right to self-determination has been identified as a “world order principle… that must be a basis of social and political organization if we are to progress along the road toward a peaceful and humane world” [15]. Self-determination includes the freedom to determine political status and pursue economic, cultural, and social development [16]. The right to self-determination for Indigenous Peoples was first recognized internationally in article three of the United Nations Declaration on the Rights of Indigenous Peoples (UNDRIP). UNDRIP expressly acknowledged distinct cultural rights for Indigenous Peoples: “By virtue of [the] right [to self-determination, Indigenous Peoples] freely determine their political status and freely pursue their economic, social and cultural development” [17]. Despite the important protection that UNDRIP offers Indigenous Peoples, Australia was one of four countries that opposed the declaration in 2007. Australia shares a history of invasion, oppression, and racism with the other countries that opposed UNDRIP: Canada, the United States, and Aotearoa/New Zealand [18].

The principles of self-determination contend that Indigenous Peoples have the capacity to establish what is of moral importance and which related political objectives they choose to prioritize; these decisions cannot be made by a guardian or assumed on behalf of Indigenous Peoples. For these reasons, we can conceptualize not the right—but the power—of self-determination, where this power includes Indigenous self-government of affairs [19,20]. Self-determination is a circumstance of political capacity wherein “by focusing on ‘everyday’ acts of resurgence, one disrupts the colonial physical, social and political boundaries designed to impede [Indigenous] actions to restore [Indigenous] nationhood” [21]. Self-determination radically opposes an assimilationist order wherein Indigenous Peoples are deliberately positioned as powerless and Indigenous aspirations are excluded [19]. As such, from a decolonial standpoint, self-determination can provide the tools to advance demands for external self-determination and anti-colonial norms, and anti-racist consciousness [22].

Despite the importance of self-determination for Indigenous Peoples, it remains difficult to find specific arrangements that ensure the survival of Indigenous cultures and institutions that operate outside of frameworks championed by states within which Indigenous Peoples live. That is, often, Indigenous Peoples are granted internal self-determination where communities participate in processes of power, rather than external self-determination, where communities are given the freedom to determine their own international status, including the power to choose political independence [15,23]. While neoliberal ideologies of personal autonomy theoretically have the potential to benefit Aboriginal and Torres Strait Islander communities, due to parallels with the notion of self-determination, the Australian state has repeatedly failed to apply this ideology to Aboriginal and Torres Strait Islander affairs, which instead remain firmly entrenched in colonial discourses and paternalistic attitudes [24]. Neoliberal rationality enables an argument that Aboriginal and Torres Strait Island Peoples fail to make ‘good’ choices at the individual level, as measured through health outcomes, while ignoring the colonial social structures and contexts of racism and disadvantage created and maintained by the state. The state continues to assert its position as knowing ‘what is best’ and neoliberalism has enabled increased authoritarianism, wherein Aboriginal and Torres Strait Islander Peoples are seen as needing benevolent white governance: “The individualism of neoliberalism informs the discourse of pathology within the race war, enabling the impoverished conditions under which Indigenous people live to be rationalized as a product of dysfunctional cultural traditions and individual bad behavior” [25].

Aboriginal and Torres Strait Islander Peoples have been intentionally and systematically locked in a paradox wherein the state deems communities as incapable of adhering to neoliberal individual responsibility, thus manufacturing a need for Aboriginal and Torres Strait Islander Peoples to be governed [26]. This authoritarianism and paternalism is enacted through political agendas, such as closing the gap, which has been described as a colonial endeavor to maintain control over Aboriginal and Torres Strait Islander Peoples within the boundaries of the nation-state [24,27,28]. Rather than encouraging self-determination for Aboriginal and Torres Strait Islander Peoples, neoliberal frameworks maintain justifications for the Australian state to control the agenda and affairs of Aboriginal and Torres Strait Islander communities. This approach sustains and encourages narrow perceptions of Aboriginal and Torres Strait Islander Peoples as dependent and passive actors rather than active decision-makers, ultimately reinforcing colonial values.

Self-determination in its purest form goes beyond personal autonomy within neoliberal economies and fundamentally includes the choice to participate within a mainstream neoliberal economy [24]. The forced acculturation and participation in the global economy have proven damaging to Indigenous well-being [12]. Corntassel has asserted the importance of understanding that self-determination represents more than just a political struggle, explaining that “resurgence means having courage and imagination to envision life beyond the state” [21]. Irrespective of the decision to participate in the Australian neoliberal economy, the Aboriginal and Torres Strait Islander political and economic agenda must be designed and controlled by Aboriginal and Torres Strait Islander Peoples in order to reflect and encompass the vast diversity of Aboriginal and Torres Strait Islander values and circumstances across Australia [24]. The power to formulate policy that meets one’s social, political, and cultural contexts is a foundational feature of self-determination [29,30]. Aboriginal and Torres Strait Islander Peoples also have the right to administer programs subsequent to policy development through their own institutions, to the extent that is possible, as is seen with ACCHS [17].

## 3. Self-Determination & Aboriginal Community Controlled Health Services

Due to the unique ability of ACCHS to support Aboriginal and Torres Strait Islander self-determination, the ACCHS model in Australia has been described as the gold standard regarding implementation of the right to self-determination enshrined in UNDRIP [27]. While Canada, the United States, and Brazil all have health services directed to meet the needs of Indigenous Peoples, these models fall short of equitably embodying the right to self-determination, with Indigenous Peoples across these countries calling for greater control over their well-being [31,32,33]. The core business of ACCHS is to reduce the health disparities between Aboriginal and Torres Strait Islander Peoples and non-Indigenous Australians, a directive supported by the Australian government [34]. The well-being of Aboriginal and Torres Strait Islander communities is vital for the well-being of individuals within a Community; Community well-being comprises overall physical, spiritual, psychological, economic, and political health [35]. Central to the maintenance of all these aspects of well-being is the fulfillment of the Aboriginal and Torres Strait Islander right to self-determination, which is championed by ACCHS. Today, there are over 140 ACCHS across Australia that are committed to putting Aboriginal and Torres Strait Islander health in Aboriginal and Torres Strait Islander hands [36]. The governance models of ACCHS prioritize Community leadership, control, and, ultimately, accountability to Community members [37]. ACCHS create space for engagement, advocacy, and employment that align with Aboriginal and Torres Strait Islander values and cultures; creating this space within a predominantly Western health system enables a shift in power dynamics that centers Aboriginal and Torres Strait Islander knowledges and values of self-determination [38].

ACCHS are consistently recognized for their success in attracting and retaining Aboriginal and Torres Strait Islander patients, as well as improving health outcomes. Through the provision of culturally responsive and comprehensive care, ACCHS reduce experiences of racism and barriers to accessing care, which progressively improves the well-being of Aboriginal and Torres Strait Islander communities [27]. ACCHS provide a cultural brokerage between biomedical conceptualizations of disease and Aboriginal and Torres Strait Islander understandings of well-being that build on familiar relationships of Community trust; both of these aspects are imperative to the comprehensive identification of patient needs and Community utilization of services [39]. The exploration of self-perceived health determinants among Aboriginal and Torres Strait Islander Peoples has identified the Community empowerment related to ACCHS as associated with improved healthcare-seeking and Community well-being [40]. Rather than utilizing a top-down approach that employs universal solutions for the ‘benefit’ of participants, ACCHS prioritize comprehensive primary healthcare or a bottom-up approach, wherein health concerns are addressed through interventions that utilize Aboriginal and Torres Strait Islander knowledge combined with appropriate professional expertise [41]. Notably, the comprehensive primary healthcare model effectively redistributes the power from the state and health professionals to Aboriginal and Torres Strait Islander communities. The continuance of ACCHS since their establishment in the 1970s, despite policy cycles and government changes, speaks to their robust and effective care provision, grounded in the right to self-determination for Aboriginal and Torres Strait Islander Peoples [27,28]. There is an opportunity to extend Aboriginal and Torres Strait Islander self-determination in health by utilizing this bottom-up approach to incorporate Community needs and decisions into national-level programs and health policies; this could also include the provision of care that meets Community needs in non-Community controlled health service settings.

## 4. Aboriginal Community Controlled Health Services as Neoliberal Resistance

The very existence and success of ACCHS in creating more equitable and emancipatory experiences of health for Aboriginal and Torres Strait Islander Peoples is a force of resistance against Australia’s neoliberal ideologies. ACCHS represent sites of ‘radical possibility’ and ‘space[s] of resistance’ [42] for Aboriginal and Torres Strait Islander Peoples due to their strength and power in prioritizing Aboriginal and Torres Strait Islander ways of knowing, being, and doing [27]. By continually demonstrating the need for Community-governed health services through successful service provision and high Community engagement, as well as through the prioritization of collectivist values and holistic care, ACCHS directly resist two of the commonly ascribed neoliberal tenets: reduced public expenditure on infrastructure and personal autonomy. ACCHS are one of the only remaining publicly funded organizations in Australia that are fully accountable to and governed by Aboriginal and Torres Strait Islander communities [27]. Further, ACCHS are Community-based and grounded in collectivist values that defy notions of personal autonomy or individual responsibility for health, instead utilizing a comprehensive primary care model that privileges Community knowledge; this form of resistance has been termed ‘Community autonomy’ [12]. While personal autonomy is central to neoliberalism, individualistic ideologies and private ownership do not align with long-held Aboriginal and Torres Strait Islander values; resisting the assumed uptake of individualism contests the expectation of Aboriginal and Torres Strait Islander Peoples to forego socio-cultural systems and fundamentally supports the right to self-determination [24]. ACCHS embody an alternative to imposed ideologies while simultaneously honoring Aboriginal and Torres Strait Islander diversity and representing collective wisdom and Community resilience [27].

While ACCHS continue to receive funding from the Australian state, the expenditure increases since the 1990s remains insufficient to overcome the disproportionate burden of mortality and morbidity experienced by Aboriginal and Torres Strait Islander communities, as well as adequate salary rates to retain Aboriginal and Torres Strait Islander staff [39,43]. Funding structures must reflect the relative and specific needs of Aboriginal and Torres Strait Islander Peoples; the continued struggle for adequate funding experienced by ACCHS represents practical constraints on the embodiment of Aboriginal and Torres Strait Islander self-determination within the Australian state. To strengthen the effectiveness of ACCHS in alignment with principles of self-determination, an equitable share of the health dollar and meaningful allocation of expenditure directed by Aboriginal and Torres Strait Islander Peoples is needed [44]. For fundamental changes regarding Aboriginal and Torres Strait Islander self-determination, the “redistribution of resources and power in the political process and the increased ability of marginalized communities to control key processes that influence their lives” [41] is required. State support for ACCHS should entail relationships of genuine trust instead of support conditional on criteria determined by the state to measure success [24]. Continued reliance on cyclical state funding restricts ACCHS priorities, structures, and the scope for self-determination; there is a clear need for ongoing state commitments and trusting partnerships based on shared values of health equity and self-determination [27].

## 5. Conclusions

In the Australian state, neoliberalism maintains colonial power and restricts the self-determination of Aboriginal and Torres Strait Islander Peoples. Dedicated funding relative to the need of Aboriginal and Torres Strait Islander communities for ACCHS as identified by Aboriginal and Torres Strait Islander Peoples is required. ACCHS must be commended for their work in resisting the power of the Australian state and the hegemonic nature of neoliberal ideologies in Australian society; the Community strength and preservation of self-determination is admirable. The power of self-determination lies in the politics of possibility; these possibilities manifest when sovereignty truly lies with Aboriginal and Torres Strait Islander authority, as is demonstrated by ACCHS [19,21]. The Australian state and society must fully comply with and respect Aboriginal and Torres Strait Islander Peoples’ right to self-determination to ensure Aboriginal and Torres Strait Islander communities have the power to overcome legacies and ongoing impacts of neoliberalism and colonization and attain equitable health outcomes.

## Data Availability

Not applicable.
